# Lung Transplantation, Pulmonary Endothelial Inflammation, and Ex-Situ Lung Perfusion: A Review

**DOI:** 10.3390/cells10061417

**Published:** 2021-06-07

**Authors:** Keir A. Forgie, Nicholas Fialka, Darren H. Freed, Jayan Nagendran

**Affiliations:** 1 Division of Cardiac Surgery, Department of Surgery, University of Alberta, Edmonton, AB T6G 2B7, Canada; kforgie@ualberta.ca (K.A.F.); dhfreed@ualberta.ca (D.H.F.); 2 Mazankowski Alberta Heart Institute, Edmonton, AB T6G 2B7, Canada; 3 Faculty of Medicine and Dentistry, University of Alberta, Edmonton, AB T6G 2R3, Canada; nfialka@ualberta.ca; 4 Alberta Transplant Institute, Edmonton, AB T6G 2E1, Canada; 5 Canadian Donation and Transplantation Research Program, Edmonton, AB T6G 2E1, Canada

**Keywords:** lung transplantation, ischemic reperfusion injury, pulmonary inflammation, primary graft dysfunction, ex-situ lung perfusion

## Abstract

Lung transplantation (LTx) is the gold standard treatment for end-stage lung disease; however, waitlist mortality remains high due to a shortage of suitable donor lungs. Organ quality can be compromised by lung ischemic reperfusion injury (LIRI). LIRI causes pulmonary endothelial inflammation and may lead to primary graft dysfunction (PGD). PGD is a significant cause of morbidity and mortality post-LTx. Research into preservation strategies that decrease the risk of LIRI and PGD is needed, and ex-situ lung perfusion (ESLP) is the foremost technological advancement in this field. This review addresses three major topics in the field of LTx: first, we review the clinical manifestation of LIRI post-LTx; second, we discuss the pathophysiology of LIRI that leads to pulmonary endothelial inflammation and PGD; and third, we present the role of ESLP as a therapeutic vehicle to mitigate this physiologic insult, increase the rates of donor organ utilization, and improve patient outcomes.

## 1. Introduction

Lung transplantation is the gold standard treatment for end-stage lung disease. It has been thirty years since Dr. Joel Cooper and his team in Toronto performed the first successful double lung transplant [1], and the field has advanced significantly since then. Approximately 60,000 adult lung transplantations were performed worldwide between 1985 and 2016 according to the Registry of International Society for Heart and Lung Transplantation (ISHLT), the foremost society on lung and heart transplantation and research [2]. Lung transplantation helps patients with end-stage lung disease due to a number of causes such as chronic obstructive pulmonary disease (COPD), cystic fibrosis, sarcoidosis, interstitial lung disease (ILD), pulmonary fibrosis, and pulmonary hypertension [2].

In spite of the accomplishments made in lung transplantation (LTx) thus far, there remains a high waitlist mortality of 40% and poor organ availability [3]. The number of waitlisted patients for lung transplantations has increased steadily over the past thirty years; unfortunately, the supply of suitable donor organs has not [4,5]. Stringent donor organ acceptance criteria along with excessive geographic distance for transportation have contributed to a low utilization rate of 20%, which is far worse than other solid organ transplants [1,2]. Donor organs that fall outside the ISHLT standard acceptance criteria, so-called “extended criteria donors”, are used by some centers to help address the high morbidity and waitlist mortality of patients [4,5]; however, the appropriate selection and management of marginal quality donor lungs is difficult. The principal concern with excessive transport times or the utilization of extended criteria donors is the increased risk of primary graft dysfunction post-operatively.

Primary graft dysfunction (PGD) is a form of acute lung injury that can occur after transplantation within the first 72-h [6]. At a tissue level, PGD is the result of diffuse alveolar damage. This damage manifests clinically as severe hypoxemia and lung edema with diffuse pulmonary infiltrates as seen on CXR. PGD is the leading cause of 30-day mortality post-LTx, affecting 11–25% of patients [7]. It also increases the risk for chronic graft dysfunction and bronchiolitis obliterans syndrome, a form of chronic allograft rejection [8]. Indeed, optimal post-operative outcomes are dependent on the prevention of PGD, which is produced by the culmination of transplant injuries with ischemic-reperfusion injury (IRI) thought to be the primary contributor [9,10,11].

IRI is tissue injury to an organ following the cessation and reestablishment of blood flow and oxygen delivery. The mechanisms of lung IRI (LIRI) occur during retrieval, transportation, and subsequent reimplantation of the donor’s lungs. It is a paradoxical injury cascade that follows reintroducing oxygen and nutrients after a period of ischemia. This deleterious cellular cascade is complex and will be discussed in detail later during this review. The extent of LIRI is positively correlated to the extent of ischemia prior to reperfusion [9,12], and the clinical signs can manifest h after transplantation as the cascade unfolds, regardless of the technical proficiency of the implantation. Endothelial cell dysfunction and disruption of the endothelial barrier are hallmarks of LIRI, contributing greatly to PGD and the inadequate supply of donor organs.

The clinical gold standard for organ preservation is cold static preservation (CSP); however, ex-situ lung perfusion (ESLP) is a relatively novel donor lung preservation and reconditioning technology that has been shown to improve lung function and increase organ utilization [13]. CSP involves storing lungs in a bag of preservation solution on ice in a cooler (4–8° Celsius). Normothermic (37° Celsius) ESLP is a means of mechanically ventilating and perfusing donor lungs under physiologic conditions. Where CSP is a black box, ESLP is an interactive, real-time evaluation and therapeutic tool that can reduce the effects of PGD caused by LIRI. Both approaches will be discussed later on. This review addresses three major topics in the field of lung transplantation: first, we review the clinical manifestation of LIRI post lung transplantation; second, we discuss the pathophysiology of LIRI that leads to pulmonary endothelial inflammation and PGD; and third, we present the role of ex-situ lung perfusion as a therapeutic vehicle to mitigate this physiologic insult, increase the rates of donor organ utilization, and improve patient outcomes.

## 2. Hostile Environments, Risk Factors, Clinical Manifestations and Treatment of Primary Graft Dysfunction in Lung Transplantation

The events preceding lung retrieval as well as those that occur during transportation and after lung transplantation, all contribute to the development of LIRI and PGD. In this sense, the logistics of lung transplantation are made up of a series of hostile environments. To better appreciate the risks to graft function during lung transplantation, it is helpful to examine the events in terms of donor-related/retrieval risk factors, transportation/CSP risk factors, and recipient/transplantation risk factors.

### 2.1. Pretransplant/Donor Risk Factors for PGD

A number of pre-transplant and donor-related risk factors are associated with LIRI and the development of PGD [14]. Studies of transplant outcomes have shown that during the first 24 h post-transplantation, donor factors predominate [15] whereas recipient risk factors are more influential beyond 24 h [16]. Donor-related risk factors for PGD include those that are pre-existing/hereditary and acquired.

Pre-existing/hereditary factors include race, sex, advanced age, and smoking [15]. Advanced age is a risk factor in other solid organ transplants, and PGD significantly increases when donors are older than 32–45 years [17]. The most significant pre-existing donor risk factor for PGD is a history of smoking [18].

Acquired donor risk factors are numerous and include excessive blood transfusions, hemodynamic instability resulting from brain death, aspiration, pneumonia, trauma, and prolonged mechanical ventilation [15]. Thromboembolism and fat embolism increase the risk of PGD by 5-fold and 25-fold, respectively [19]. Clinical factors that often precede brain death such as loss of surfactant, platelet occlusion of the microvasculature during hypovolemic states, pro-inflammatory cascades, and upregulation of adhesion molecules all negatively affect pulmonary endothelial function, which is the primary target of LIRI [20].

A deleterious cascade of events surrounding brain death contributes to negative changes in the pulmonary vasculature. Braindead donors, or neurologic determination of death (NDD), donors are the most common donor for LTx. Brain death results in parasympathetic and sympathetic imbalance. This causes the release of inflammatory cytokines, hemodynamic instability with tachycardia, hypothermia, electrolyte derangements, and endocrine perturbations [15]. Donor hypotension and microvascular occlusion impair organ perfusion causing ischemia and compromising graft quality [21]. These effects are compounded by poor tissue oxygenation during warm and cold ischemia at the time of retrieval, triggering cell dysfunction, necrosis, and apoptosis [22]. Braindead donors are also prone to pulmonary injury from mechanical ventilation-induced barotrauma and alveolar recruitment maneuvers, despite the use of protective ventilation strategies. In sum, there are a number of consequences to graft quality as a result of brain death and the events preceding organ retrieval.

### 2.2. Procurement and Cold Static Preservation: Gold Standard Preservation Strategies Contribute to Primary Graft Dysfunction

Explantation of the donor lung necessarily involves the complete cessation of blood supply (ischemia) and ventilation (hypoxia), and clinicians employ the use of cold temperatures and preservations solutions to mitigate the resulting injury. The complete cessation of oxygenation and blood supply is known as anoxic ischemia. This is characteristically different from ventilated ischemia, such as following a pulmonary embolism, where blood flow is interrupted but ventilation continues [23,24,25]. Therefore, lung retrieval and the associated LIRI are due to anoxic ischemia [26].

To limit the extent of LIRI during retrieval, surgeons employ several protective surgical and storage strategies. The most rudimentary strategy is an efficient operation that limits the duration of warm ischemia when the lungs are being surgically divided from their associated structures, including the heart and trachea. Prior to this step, most institutions flush the lungs antegrade through the pulmonary artery with a hypothermic, low-potassium, and dextran-containing solution. This solution composition has been established to produce superior graft function in animal transplantation models [27]. The total volume of the flush is around 60 mL/kg with PA pressures of 10–15 mmHg. Heparin and prostaglandin are administered ahead of the flush to prevent clot formation, decrease inflammation, and limit vasoconstriction and are associated with hypothermia and hypoxia [10,28]. Depending on the center an additional retrograde flush is often performed to remove any residual blood and clots. The lungs are clamped at moderate expansion (approximately 50% of total lung capacity) to maintain alveolar recruitment, optimize surfactant production, and provide a source of oxygen (albeit limited) during transportation [10,15,28]. Lungs are stored in a plastic bag containing additional preservation solution, then placed in a cooler full of ice. This strategy preserves the lungs at a temperature between 4 °C and 8 °C, which is considered protective for up to eight h. Preservation beyond eight h increases the risk of PGD significantly [10,15,28]. For this reason, ischemic durations beyond 8 h are a limitation to successful transplantation and must be factored into consideration prior to a transplant decision [10]. The aforementioned efforts to protect the lungs are helpful, but they do not protect the lungs from injury during the reperfusion phase at the time of transplantation [14].

### 2.3. Posttransplant/Recipient Risk Factors for PGD

There are a number of post-transplant and recipient-related risk factors associated with LIRI and PGD. Again, risk factors can be categorized as pre-existing/inherited and acquired. Pre-existing risk factors are similar between donors and recipients and include age, gender, and race [29,30]. Acquired recipient risk factors include elevated body mass index, pulmonary hypertension, idiopathic pulmonary fibrosis, sarcoidosis, underlying liver, and kidney disease, and heart failure, which are all associated with increased risk of PGD postoperatively. Elevated BMI portends the greatest increased risk among acquired risk factors, with BMI > 30 associated with an absolute risk increase of 11% for PGD compared to normal BMI controls [16,17,29].

Certain risk factors are associated with the transplantation surgery itself including the use of transfusions, elevated pulmonary artery pressures, and excessive mechanical ventilation. Transfusion-related lung injury (TRALI) is a known risk factor for PGD postoperatively resulting from intraoperative blood transfusions [30]. The manner in which reperfusion is carried out also influences PGD development. Research has demonstrated that pulmonary artery pressures should be increased gradually during reperfusion, particularly during the first ten minutes; hence, after completion of the vascular anastomosis, the PA clamps should be opened in phases over a ten-minute period or longer [31,32,33]. When cardiopulmonary bypass (CPB) is used for transplantation, the flow can be gradually increased in a similar fashion from the pump. As in the donor operation, mechanical ventilation can injury the lungs or exacerbate underlying injury leading to ventilator-associated lung injury and PGD [34]. For example, high tidal volumes and low positive end-expiratory pressure (PEEP) actually worsens lung function three h after reperfusion [35]. A common protective ventilation strategy is to gradually ventilate the lungs prior to unclamping the PA by targeting an FiO_2_ of 50%, PEEP of 5 cm H2O, and peak pressure of 20–25 cm H2O [36]. Recipient fluid overload, infection, and post-operative hemodynamic deterioration are also associated with PGD. These strategies are beneficial but do not eliminate the risk of PGD postoperatively.

### 2.4. Clinical Manifestation of PGD Post LTx

The hallmark clinical features of PGD are poor oxygenation and diffuse pulmonary infiltrates on chest X-ray within 72 h post-transplantation [10]. Related features of pulmonary function are likewise worsened such as decreased pulmonary compliance, increased pulmonary vascular resistance, and the development of intrapulmonary shunts [37]. A rise in peak airway pressures makes adequate ventilation challenging. The combination of increased PVR and increased vascular permeability results in variable degrees of noncardiogenic pulmonary edema. In turn, there is progressive V/Q mismatch and poor oxygenation. In this way, PGD is similar to Acute Respiratory Distress Syndrome (ARDS) with regards to histology and clinical features [38,39]. PGD manifestations exist on a continuum of severity, which has been established by the ISHLT. Still, the diagnosis remains one of exclusion without specific diagnostic criteria [40].

PGD severity is qualified by the extent of hypoxemia with reference to the administered FiO_2_ (PaO_2_/FiO_2_ ratio) (Table 1). LIRI and PGD are associated with adverse outcomes including prolonged mechanical ventilation, increased post-operative length of stay, and increased mortality [20,41,42]. The presence of stage 3 PGD within 72 h is strongly associated with poor outcome [43]. This is in part related to the systemic consequences of LIRI that reach beyond the lungs and result in multisystem organ dysfunction.

PGD severity is qualified by the extent of hypoxemia with reference to the administered FiO_2_ (PaO_2_/FiO_2_ ratio) (Table 1). LIRI and PGD are associated with adverse outcomes including prolonged mechanical ventilation, increased post-operative length of stay, and increased mortality [20,41,42]. The presence of stage 3 PGD within 72 h is strongly associated with poor outcomes [43]. This is in part related to the systemic consequences of LIRI that reach beyond the lungs and result in multisystem organ dysfunction.

### 2.5. Postoperative Care and Current Treatment of Lungs with PGD

At present, there is no specific treatment for the consequences of LIRI and PGD, only supportive therapy to allow time for recovery and limit secondary damage. Paradoxically, reperfusion of the ischemic organ is the required treatment, which simultaneously triggers the cascade of events that can lead to organ compromise. Supportive measures include the use of protective ventilation with permissive hypercapnia, moderate to high PEEP (8–10 cm H_2_O), low tidal volumes (6–8 mL/kg), and low peak inspiratory pressures (≤30 cm H_2_O) to avoid overdistension of alveoli [45]. Judicious fluid management to avoid fluid overload, and the optimization of hematocrit (25–30%) along with coagulation parameters are also targeted [46]. In cases resistant to standard supportive measures, inhaled nitric oxide can help lower pulmonary artery pressures and correct ventilation-perfusion mismatch [47]. Extracorporeal membrane oxygenation (ECMO) can also be employed as a final lifesaving measure. ECMO can help protect the lungs from aggressive ventilatory requirements for oxygenation goals while mitigating the harmful systemic effects of hypoxia [48,49]. Given the severity of repercussions from LIRI and PGD, it is important to further understand the pathophysiology to guide research, prevention, and treatment.

## 3. Pathophysiology of Ischemic Reperfusion Injury, Pulmonary Endothelial Inflammation, and Primary Graft Dysfunction

LIRI leads to cellular and molecular dysfunction that causes pulmonary inflammation, edema, and subsequent primary graft dysfunction. Broadly speaking, ischemia and subsequent reperfusion of the lungs causes the production and release of reactive oxygen species (ROS), activation of sterile immunity (innate and adaptive immunity), release of inflammatory cytokines and downstream signaling cascades, altered metabolism, and derangement of ionic homeostasis [50]. In turn, these deleterious events trigger apoptosis and necrosis of pulmonary cells as well as damage to the pulmonary endothelium and alveolar epithelium. This results in increased fluid permeability to the lungs, the development of pulmonary edema, and impaired oxygen exchange [41,51,52]. Diffuse pulmonary infiltrates on CXR from fluid accumulation in the lungs along with impaired oxygenation due to poor lung function are the hallmarks of PGD, as previously discussed. Each of the aforementioned processes is highly complex and functions not so much in a linear fashion that culminates in PGD, but more so as overlapping amplification events such as in a feedforward cycle.

**Figure 1:**
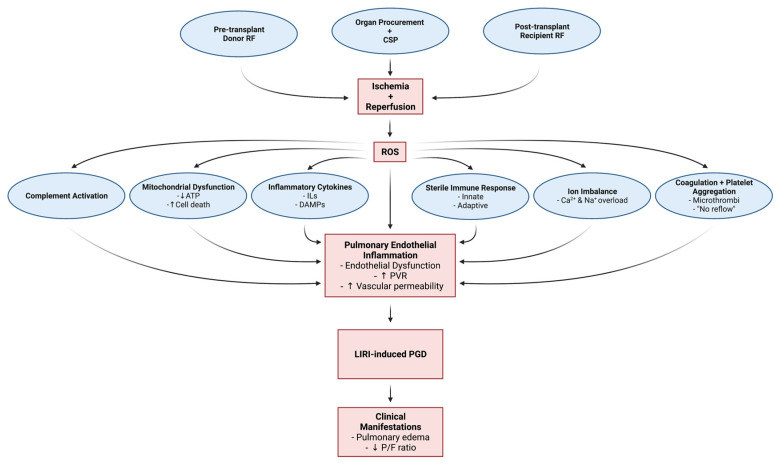
Factors and cellular mechanisms associated with lung ischemic reperfusion injury and primary graft dysfunction in lung transplantation. Risk Factors, RF; Cold Static Preservation, CSP; Reactive Oxygen Species, ROS; Adenosine Triphosphate, ATP; Interleukins, ILs; Damage Associated Molecular Patterns, DAMPs; Pulmonary Vascular Resistance, PVR; Lung Ischemic Reperfusion Injury, LIRI; Primary Graft Dysfunction, PGD; PaO_2_/FiO_2_ ratio, P/F ratio. Created with BioRender.com.

Reperfusion is a necessary evil to protect the cells from certain death, but paradoxically triggers a deleterious cascade of tissue injury that is proportional to the extent of ischemia and can be fatal [52,53,54,55]. In the past, it was believed that the lungs were resistant to IRI due to their dual blood supply (pulmonary and bronchial arteries) and increased availability of oxygen through ventilation; however, ample research, particularly in transplantation, has established that lungs are indeed highly susceptible to IRI [10,26]. Figure 1 is a schematic representation of the pathophysiology of LIRI. Extensive research has revealed some of the underlying mechanisms involved in LIRI; however, ongoing research is still needed to clarify the signal transduction pathways and identify specific treatments. To better understand the complexities of LIRI, this review will attempt to isolate and summarize the overlapping pathophysiologic and cellular mechanisms.

### 3.1. Pathophysiologic and Cellular Mechanisms of LIRI, Pulmonary Inflammation and PGD

#### 3.1.1. Oxidative Stress Response: Reactive Oxygen Species Generation and Downstream Signaling

ROS generation is theorized to be the inciting event in LIRI, particularly at reperfusion [56]. Subsequent downstream signaling responses along with parallel processes produce a feedforward mechanism resulting in greater ROS production, which further exacerbates and amplifies tissue injury [55,57]. ROS are highly reactive free oxygen radicals, hydroxyl radicals, and nonradical molecules that are responsible for the activation of many physiological signaling cascades [58,59,60]. Examples of ROS include superoxide anions, hydrogen peroxide, and hydroxyl radicals, which are the most unstable and reactive. The observation that LIRI is initiated following the reintroduction of oxygen at the time of reperfusion led to the theory of ROS generation as the key signaling event [56]. Following reperfusion, the rate of ROS generation exceeds the rate of ROS clearance [57]. Specifically, ROS production increases in macrophages, endothelial cells, vascular smooth muscle cells, and alveolar type II cells [51,61]. The elevated level of ROS leads to increased activation of apoptosis [62], intracellular calcium overload [63], and innate immune responses [64]. Normally, compensatory antioxidant mechanisms in the body helps balance ROS levels; however, reperfusion overwhelms these protective processes [10,51,65,66,67]. Although the production of ROS increases drastically during reperfusion, there is considerable evidence that ROS generation increases prior to reperfusion during the ischemic phase [63,68,69]. ROS are produced from dysfunctional mitochondrial electron transport using residual oxygen present during the ischemic period [70]. Upon reperfusion, waste products, such as hypoxanthine and succinate, are metabolized to generate ROS [71,72]. ROS then triggers downstream activation and production of inflammatory cytokines, sterile inflammation, and the development of ionic derangements.

#### 3.1.2. Inflammation: Cytokines, Chemokines, and Damage-Associated Molecular Patterns (DAMPs)

The rapid and complex inflammatory response induced by LIRI causes the release of cytokines and damage-associated molecular patterns (DAMPs) along with a robust immune response (discussed later). The vigorous generation of ROS is largely responsible for initiating these cascades leading to the activation of multiple cell types, lipid membrane peroxidation, and secretion of inflammatory cytokines and DAMPs causing tissue injury and cell death [56]. Early on following reperfusion, there is an increase in pro-inflammatory molecules such as IL-1Beta, IL-2, IL-8, IL-12, IL-18, TNF-alpha, and IFN-gamma, which leads to tissue damage, necrosis of pulmonary cells, and ultimately pulmonary graft dysfunction [10,73,74,75,76]. This influx of cytokines also contributes to platelet aggregation, coagulation, and vascular dysfunction (discussed later). DAMPs are a group of proinflammatory mediators activated during LIRI. These include high-mobility group box 1 (HMGB1), fibronectin, heat shock proteins, and oxidized phospholipids to name a few [77]. DAMPs mediate their effects via pattern recognition receptors (PRRs) including toll-like receptors (TLRs) that have been implicated in LIRI. Specifically, the mammalian lipopolysaccharide receptor TLR4 is thought to be a key modulator of LIRI [78] and sterile inflammation [79] (discussed later). Research has demonstrated that inflammatory lung injury in LIRI is mediated by TLR4 activation and downstream signaling via nuclear factor (NF)-kappabeta [77]. Additional studies have used plasma biomarkers and genetic analysis to demonstrate the role of TLRs and the innate immune response in PGD [80,81]. The flux of pro-inflammatory cytokines and DAMPs drives tissue injury and the activation of sterile immune responses via innate and adaptive mechanisms [82,83,84] in a number of cells such as endothelial cells, alveolar type II cells, vascular smooth muscle cells, and resident macrophages [85,86].

#### 3.1.3. Sterile Immunologic Injury: Innate and Adaptive Immune Responses

Sterile inflammation occurs following trauma and other non-infectious forms of injury, such as IRI [79]. Sterile inflammation, also referred to as sterile immunity, involves locoregional immune responses that mirror the host response to microbial invasion. Indeed, both innate and adaptive immune mechanisms are activated in zones of sterile injury and cell death [53]. The innate immune response is the first to be activated, which leads to the infiltration of alveolar and extravascular spaces by recipient polymorphonuclear neutrophils (PMNs) [82], the activation of invariant natural killer T (iNKT) cells, and activation of alveolar macrophages [5]. During this period there is an increase in the production of pulmonary cell and macrophage adhesion molecules [10,73]. Cellular adhesion molecules (CAMs) enhance the binding of PMNs to endothelial cells [87]. Activation of neutrophils, their adherence to and infiltration of the donor’s lung, along with other innate immune cells, are well established in the literature [85,88,89,90,91,92]. Donor lung macrophages, epithelium, and endothelium produce and activate chemokines, specifically IL-8 and C Motif Chemokine Ligand 2 [CXCL2] [93], that propel the infiltrative processes seen in LIRI. Indeed, the recruitment and adherence of PMNs to the vessel wall appear to emanate from the inflamed endothelial layer of donor lung vessels. The transmigration of activated leukocytes into the extravascular and alveolar spaces causes microvascular permeability via toxic release of ROS, proteases, elastases, and endothelial gap formation [94] (discussed later). The initial innate immune response is thought to be caused by a robust production of ROS upon reperfusion, and the activation of alveolar macrophages [83] triggers the further release of inflammatory cytokines resulting in further neutrophil recruitment and ROS generation [95,96]. In this sense, ROS generation is self-perpetuating in LIRI via cytokine release and a sterile immune response that produces progressively worse pulmonary dysfunction and further ROS production [64]. The adaptive immune system is also activated by IRI, although it is not as well understood. Research has established that T-cells are activated via antigen-dependent and -independent manners [53,97]. Cellular immunity against the ischemic organ follows from danger signals released from the ischemic tissue, which leads to the presentation of cellular antigen [97]. B-cells are also activated during IRI by neoepitopes expressed by ischemic cells, leading to antibody-mediated tissue injury [98,99,100]. Inflammatory and immunological injury are critical processes of LIRI and PGD.

#### 3.1.4. Multifactorial Cellular Mechanisms: Anaerobic Metabolism, Ion Imbalances and Mitochondrial Dysfunction

LIRI is caused by multifactorial cellular mechanisms [26,56]; this includes conversion to anaerobic metabolism, development of ionic imbalances, and progressive mitochondrial dysfunction. Depletion of oxygen results in a decrease in oxidative phosphorylation, a drastic decrease in ATP concentration [101], and a conversion from aerobic to anaerobic metabolism [94,101,102]. Products of ATP degradation, such as hypoxanthine, accumulate and serve to produce more ROS [55,71]. Lactate accumulates during anaerobic metabolism, which decreases cellular pH [101,102]. Acidity has a direct toxic effect on the cell [103], and the increased concentrations of hydrogen ions cause sodium to accumulate intracellularly due to the activity of Na+/H+ exchangers [104]. Increased intracellular sodium attracts water and causes cells to swell [10]. Ischemia also drives an increase in intracellular calcium along with sodium [64,104] through the dysfunction of ATP-dependent cellular ion pumps [56,94]. Increases in intracellular calcium, sodium, and water contribute to cellular dysfunction [95,104]. The increased concentration of intracellular calcium causes vasoconstriction and degradation of membrane phospholipids due to the activation of several calcium-sensitive enzymes [61]. Increased vasoconstriction [105], changes in cellular shape [64], formation of endothelial gap junctions [106], and the triggering of apoptosis [107] all result from increased intracellular calcium and contribute to the development of microvascular permeability. Ion imbalance also negatively affects mitochondria, leading to swelling and apoptosis [56]. Specifically, proapoptotic factors can be released from mitochondria following an increase in mitochondrial calcium levels due to the opening of mitochondrial transition pores and swelling followed by rupture of the mitochondrial membrane [50]. Reperfusion with oxygen delivery works to normalize pH and clear toxins through wash-out, but it also initiates a related cascade of damaging events as previously outlined.

#### 3.1.5. Complement Activation, Coagulation, and Increased Platelet Aggregation

The inflammatory response of LIRI activates the complement system, and coagulation cascade with increased platelet aggregation, which together increase microvascular permeability and progressive PGD. Activation of complement pathways, in turn, activates the innate immune response that leads to further tissue damage as previously mentioned [108,109]. The activation of complement increases endothelial vascular permeability through the generation of anaphylatoxins (C3a and C5a) [110] along with the formation of membrane attack complexes (C5b-9) [111,112]. As previously mentioned, circulating cytokines released during LIRI also initiate platelet aggregation and coagulation [26,56,61]. Hypoxia creates a physiologic environment vulnerable to irregularities in blood flow by activating endothelial cells and coagulation [73]. Activated platelets contribute to pulmonary edema formation in PGD in several ways: activated platelets release vasoactive mediators including ROS, serotonin, platelet-activating factor, and thromboxane A2; furthermore, platelets bind to the pulmonary endothelium, which causes microthrombus formation, microvascular constriction, and leukocyte adhesion that contributes to neutrophil infiltration [26,56,61,113,114].

#### 3.1.6. Endothelial Cell Dysfunction, Pulmonary Vascular Resistance, and Vascular Permeability

The pulmonary vascular endothelium is the principal target of LIRI, and increased permeability of the alveolar-capillary barrier is the primary cause of pulmonary edema, which results in impaired oxygenation. Hypoxia alters the transcriptional programming of endothelial cells resulting in the downregulation of the vasodilators nitric oxide synthase and prostacyclin [94]. This coincides with the upregulation of platelet activation factor thromboxane A2 and the vasoconstrictor endothelin-1, producing an increase in smooth muscle contraction and a rise in pulmonary vascular resistance (PVR). Endothelin-1 stimulates the release of cytokines from monocyte and macrophages that causes further infiltration of the lungs as already discussed [115]. This can result in a “no re-flow phenomenon” due to decreased microvascular flow in the setting of reperfusion, contributing to prolonged lung dysfunction post-transplant [21,53,86]. In addition to “no re-flow” in certain areas of the lung, the opposite is also true, where the cardiac output is redistributed to produce areas of compensatory over-circulation. The increased hydrostatic pressure in those capillary beds promotes extravasation of fluid, causing edema [116]. Both arterial and venous vessels appear to contribute to the development of pulmonary edema in LIRI through increased PVR [116,117]. Furthermore, increased permeability allows large proteins to extravasate, thereby compromising the oncotic pressure in favor of edema formation and intravascular fluid loss [116]. Pulmonary edema in LIRI is thus a result of alterations in transcapillary hydrostatic pressures, osmotic forces, and increased permeability. Impaired gas exchange results from pulmonary edema and ventilation-perfusion mismatching from microvascular dysfunction with heterogeneous blood flow distribution, thus producing systemic hypoxemia despite reperfusion [10,26,56,118]. Lastly, the alveolar epithelium is also damaged with increased permeability and loss of its ability to clear alveolar fluid, which accumulates and exacerbates edema development [119]. In contrast to the significant amount of research regarding the effects of LIRI on pulmonary endothelium, there is a paucity of research specific to the damage upon the alveolar and bronchial epithelium. Likewise, pulmonary endothelial damage due to LIRI has been researching extensively while the effects on bronchial circulation and endothelial function are largely absent in the literature. Lack of research into the effects of LIRI in these specific tissues is a knowledge gap that requires further attention.

#### 3.1.7. Summary

In summary, LIRI results in pulmonary endothelial inflammation that underlines PGD via the following predominant mechanisms: (1) ROS generation through reperfusion (2) cytokine generation that propagates an inflammatory chain reaction, (3) compounding innate and adaptive immune system activation, (4) ion imbalance that distorts structural integrity and cell function, (5) activation of apoptosis and necrosis pathways, (6) activation of destructive complement pathways and disposition for microvascular thrombus formation, (7) endothelial cell layer gap formation, and (8) increased pulmonary vascular resistance (PVR).

## 4. Ex-Situ Lung Perfusion for the Prevention and Treatment of PGD

Research into preservation strategies to decrease the risk of PGD are needed and ex-situ lung perfusion is the foremost technological advancement in this field. Annually, rates of lung transplantation are increasing along with the average age of recipients [120]. Post-transplantation complications continue to be a major concern [11,80,121,122]. As previously mentioned, PGD due to LIRI has a reported incidence of 11-57% following LTx with a significant impact on survival outcomes [11,123]. PGD is also associated with increased short- and long-term morbidity, including the development of bronchiolitis obliterans syndrome (BOS) [18,42].

BOS is the clinical manifestation of underlying irreversible airway narrowing and is caused in part by LIRI and PGD [89]; therefore, reducing rates of LIRI and PGD by protecting the donor pulmonary and bronchial endothelium through ESLP may provide long-term clinical benefits. BOS is the leading cause of long-term morbidity, decreased quality of life, and mortality post-LTx [124,125]. BOS is most commonly caused by obliterans bronchiolitis, the progressive narrowing of the bronchioles via fibro-obliteration; however, other causes can produce the clinical effects of BOS, such as chronic rejection [125,126,127,128]. For this reason, chronic lung allograft dysfunction (CLAD) is a more encompassing term for post-transplant airway dysfunction, whereas BOS specifically refers to the most common cause of CLAD, which is obliterative bronchiolitis [129,130]. Clinically, BOS patients present with progressive worsening of lung function as measured by PFTs with a decrease in FEV1 measurements [130]. This syndrome develops in 30–50% of LTx patients within 3–5 years after surgery [131]. CLAD and BOS represent significant limitations to the enduring success of LTx. Treatment with high-dose steroids, ATGAM, and OKT3 therapy are largely unsuccessful at controlling its progression [125,132,133]. The precise mechanism of post-inflammatory fibrosis is not well established; however, studies of LIRI after LTx have demonstrated an increase in inflammatory mediators within the lung allograft [127,133], which may, in turn, upregulate the host immune response.

Technology that protects the pulmonary endothelium is essential to prevent these complications. ESLP of donor lungs has proven to decrease levels of inflammation, increase the donor pool, expand the geographic reach for transportation of organs, and recondition extended criteria lungs suitable for transplant [13,134,135,136,137,138,139,140,141,142]. ESLP also serves as a unique tool of investigation that allows for an improved understanding of the pathological processes of LTx.

### 4.1. Overview of ESLP

Normothermic (37° Celsius) ESLP is a means of mechanically ventilating and perfusing donor lungs under physiologic conditions that improve organ quality and prolongs preservation compared to CSP [11]. The standard setup for ESLP involves the following steps. The donor lungs are intubated via the trachea and attached to a ventilator. The lungs are ventilated at a respiratory rate of approximately 10–20 breaths per minute with an FiO2 of 12–50%. The pulmonary trunk is cannulated and perfused with a solution to assist in preservation and reconditioning. The perfusate solution supports metabolism with varying compositions depending on the specific protocol being followed [134,142,143,144,145,146,147]. The perfusate drains from the left atrium into a circuit for filtration and deoxygenation before returning to the pulmonary artery to complete its course. The perfusate is propelled by a centrifugal pump, similar to cardiopulmonary bypass. The circuit includes an arterial line filter to capture debris and air bubbles (particulate filter). Some circuits include leukocyte filters. It also includes a membrane oxygenator (with a built-in heater-cooler) that functions as a deoxygenator with the addition of a standard sweep gas mixture (89% N_2_, 8% CO_2_, 3% CO_2_). The sweep gas replicates systemic oxygen demand.

The perfusate biochemistry is representative of the donor lung tissue function and allows for facile assessment of organ stability. Arterial blood gas samples are routinely assessed from the perfusate circuit, informing users of acid-base status, electrolyte concentrations, and quality of oxygenation/ventilation status. Where CSP is a black box, ESLP is an interactive, real-time evaluation and therapeutic tool. It greatly increases user control of donor organ quality during transportation and helps mitigate ischemic reperfusion injury.

Although CSP remains the gold standard method for organ preservation, ESLP is becoming the superior preservation strategy, and many researchers have contributed to improving its methodology. A noteworthy contribution was by Dr. Stig Steen and his team in 2001 when they described the first human transplantation of a donor lung reconditioned on ESLP [13]. This was particularly triumphant as the lungs came from a DCD donor—a person who has died from circulatory death as opposed to the neurologic determination of death (NDD/brain death). DCD lungs were previously shown to be viable for transplantation; however, there was a need to accurately assess them prior to transplantation, which was the benefit provided by ESLP. This clinical development spurred research in the field of ESLP as a means of expanding the organ donor pool by using DCD lungs. Since then, numerous studies have shown that normothermic ESLP produces less edema, superior alveolar-epithelial tight junction integrity, better metabolic function, and improved oxygenation compared to CSP [136,138,139,140].

### 4.2. ESLP: LIRI and Pulmonary Inflammation

ESLP has been shown to decrease the extent of LIRI and pulmonary inflammation compared to CSP. ESLP use in transplantation has resulted in a global increase in lung transplants worldwide [148]. Portable ESLP machines reduce cold ischemic times and perfuse/ventilate the lungs at normothermic temperatures during transport. The use of a buffered perfusate solution and pharmacological mediators of inflammation reduces the overall inflammatory insult on lungs incurred from retrieval. ESLP has been demonstrated to reduce the severity of inflammation in donor lungs post-ischemic reperfusion by decreasing allorecognition, infiltration, and priming of recipient T cells [149]. This in part explains why ESLP contributes to reconditioning of lungs and improved graft quality at transplantation.

#### ESLP: Pulmonary and Bronchial Circulation

There are two sources of blood supply in the lungs – the pulmonary and bronchial vessels and part of the benefit of ESLP may be due to the retrograde perfusion of the bronchial arteries with high oxygen content perfusate. The lungs are predominantly perfused by the pulmonary arteries that branch from the right ventricle. These arteries are low-pressure systems of hypoxic blood. The bronchial arteries branch off of the thoracic aorta (and occasionally the intercostal arteries and coronary arteries), supplying high pressure, high oxygen content blood to the bronchial walls and surroundings structures. Although the bronchial arteries only receive approximately 1% of the cardiac output due to their small diameter [150,151,152], these vessels serve an important function, and their absence can lead to the ischemic necrosis of airway mucosa. The two circuits coalesce in capillary beds (bronchopulmonary anastomoses) near the alveolar ducts [153]. The capillary beds drain into the pulmonary veins to join the left heart. The bronchopulmonary anastomoses allow for the mixing of systemic and pulmonic blood. This continuity also serves as a protective structure against ischemia in the event of an obstruction to flow in either circuit (flow through either system can help perfuse the other). The oxygen-rich alveoli and dual circulation of the lungs provide a greater degree of protection from ischemia compared to other solid organs [26]. Unlike other solid organ transplantation, LTx is unique in that it does not reestablish systemic circulation as the bronchial arteries are not re-anastomosed [154]. This is because the technique for bronchial artery revascularization has been unreliable clinically [155]. Therefore, much of the donor bronchus viability is the result of retrograde perfusion through the bronchopulmonary capillary beds by the pulmonary arteries [156]. During CSP, the pulmonary arteries are likely more resistant to the ischemia due to their typically hypoxic circulation, whereas bronchial arteries are systemic vessels, used to systemic pressures and high oxygen contents and may be more severely affected. ESLP provides retrograde perfusion of the bronchial arteries and their surrounding structures via highly oxygenated flow through the pulmonary vessels. This protective situation may account for some of the improved graft performance and decreased inflammation seen in lungs managed by ESLP compared to CSP.

### 4.3. Negative Pressure Ventilation (NPV) versus Positive Pressure Ventilation (PPV) ESLP

**Figure 2:**
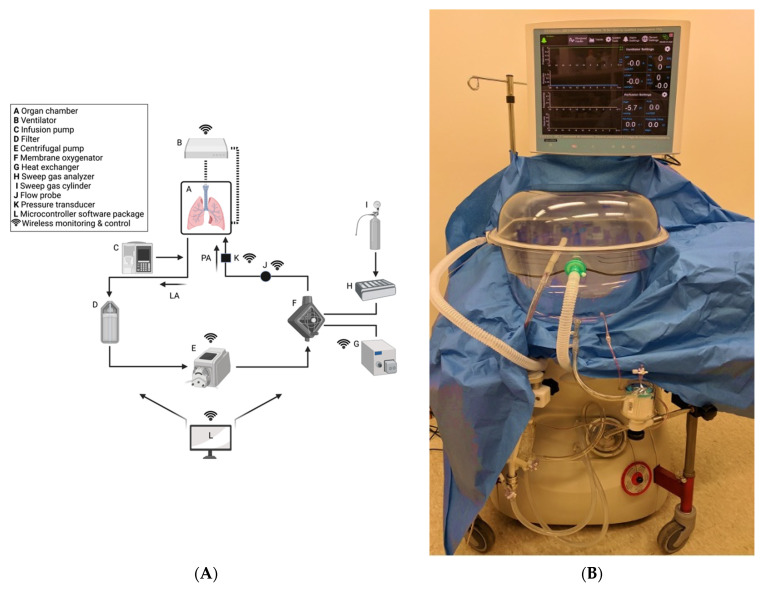
Custom-built NPV-ESLP platform: (**A**) Schematic of NPV-ESLP components; (**B**) Tevosol Inc. clinical trial prototype. (**A**) Oxygenated perfusate drains from the open left atrial system into a hard-shell reservoir (A), then through an arterial/particulate filter (D), pumped via the centrifugal pump (E) to the oxygenator/heat exchanger (F,G); which in turn warms the perfusate to normothermia (computer-controlled/adjusted (L)) and deoxygenates it with a sweep gas mixture (H,I). Prior to re-entering the lungs via the pulmonary arterial cannula (PA) for re-oxygenation, the perfusate passes by a flow probe sensor (J) and a pressure transducer (K). Negative Pressure Ventilation (NPV) circuit is depicted (B). An infusion pump (C) infuses insulin, dextrose, volume, and medications as needed.

NPV ESLP has been shown to produce improved outcomes compared to PPV ESLP in animal models [142]. There are two ventilation strategies employed in commercially available ESLP platforms: negative pressure ventilation and positive pressure ventilation. The Ex-vivo Organ Support System (EVOSS) is the only platform for humans that currently uses negative-pressure ventilation (Figure 2). This means that the EVOSS more closely replicates physiologic respiration—ventilation is achieved by pulling the lungs open with an extrapleural vacuum rather than forcing the lungs to expand by exclusively inflating them with air. In 2018, Aboelnazar et al. demonstrated that NPV-ESLP is associated with reduced inflammation and lung injury compared to PPV, irrespective of use with cellular or acellular perfusate [142]. The team also determined that human lungs lost weight during the NPV run with cellular perfusate, suggesting a “drying out effect” with this combination of ESLP strategy. Where edema is that hallmark of LIRI, the associated decrease in inflammatory markers and weight gain following NPV suggests its superior ability at mitigating LIRI.

EVOSS was developed by Dr. Darren Freed and Dr. Jayan Nagendran in 2016 at the University of Alberta in Edmonton, Canada. The aim was to develop a form of ESLP that reduced the incidence of ventilator-induced lung injury (VILI)—a mechanically induced pulmonary edema caused by high respiratory pressures and a documented consequence of prolonged PPV-ESLP [157,158,159]. A recent clinical trial demonstrated that NPV is safe when used to recondition extended criteria donor lungs from transplantation [160].

### 4.4. NPV-ESLP and Extended Criteria Donors

In 2020, Buchko et al. established the clinical safety and feasibility of EVOSS technology through a single-center clinical trial of NPV-ESLP whereby twelve extended criteria donor lungs were successfully reconditioned and transplanted into recipients, resulting in 100% 30-day and 1-year survival [160]. Recruitment took place between October 2018 to July 2019. Extended criteria donors were procured in the standard fashion with CSP during transportation to the implanting hospital. Lungs were then connected to EVOSS for preservation, reconditioning, and evaluation. If the lungs were deemed acceptable for transplantation, they remained on the NPV-ESLP until the first recipient lung was explanted. The average ESLP run was 182 min, and total cold ischemic time was approximately 308 min and 359 min for the right and left lungs, respectively. The average total time from donor explant to implantation was 8 h 14 min and 9 h 6 min for right and left lung, respectively. The mean P:F ratio was 492 and all organs met the criteria for utilization, including stable hemodynamics and oxygenation after 3 h of NPV-ESLP. In addition to the excellent survival outcomes from this trial, no patients developed PGD scores grade 3 at 72 h or required extracorporeal membrane oxygenation (ECMO) post-operatively. This study demonstrates very promising results for the commercial use of EVOSS. To further establish the clinical utility of this technology a multi-center clinical trial is required, which will be the horizon for the EVOSS team.

### 4.5. ESLP as a Therapeutic Vehicle

ESLP also provides a means of delivering targeted therapies to the donor lungs, which has multiple advantages. First, the treatments can be delivered to the lungs in isolation, which avoids any potential negative impact on other organ systems and promotes a concentrated treatment exclusive to the lungs. Second, the continual evaluation of the donor lung function on ESLP enables real-time assessment of cause-and-effect. Third, because the lungs are treated in isolation, the effects seen in animal models can more easily be translated into clinical trials [14]. At present, gene therapies, stem cell therapies, receptor agonists, and inhaled agents are being used as specific ESLP treatments for animal models of lung transplantation.

### 4.6. Animal Models of ESLP Therapeutics

#### 4.6.1. Gene Therapy

ESLP allows for the delivery of targeted gene therapies using viral vectors. For example, inflammation has been reduced in donor lungs following treatment with IL-10 gene therapy via ESLP in large animal transplant survival models and nontransplant, damaged human lung models to achieve transplant acceptable parameters [161,162,163]. In another large animal model of gene therapy via ESLP, adenoviral IL-10 decreased evidence of allograft rejection and was associated with improved lung function when compared to controls or CSP alone [161].

#### 4.6.2. Mesenchymal Stem Cells

Mesenchymal stem cells (MSC) delivery via ESLP is being studied for its therapeutic potential to reduce the signs of LIRI. Stone et al. (2017) have shown in a DCD murine model that MSC delivered to donor lungs via EVLP demonstrate improved edema and lower levels of neutrophil infiltration compared to controls. They also examined the BAL fluid and observed decreased inflammatory markers IL-17, TNF-α, CXCL1, and HMBG1 levels [164].

#### 4.6.3. A2AR Agonists

Selective adenosine 2A receptor (A2AR) agonists have been shown to improve lung parameters in transplantation models when administered via ESLP. In 2015, Stone et al. investigated the role of adding A2AR to ESLP perfusate in a murine model of lung donation compared to ESLP alone. Their findings demonstrated lower levels of inflammatory markers, including CXCL1, CCL2, and TNF-α, decreased concentration of neutrophils, lower PA pressures, and improved lung compliance [165].

#### 4.6.4. Inhaled Agents

Certain inhaled agents have shown beneficial results on lung function when used in conjunction with ESLP. For example, sevoflurane, a volatile anesthetic, has been shown to provide significant protection from LIRI in a rat model of DCD lung donation with reconditioning by ESLP. Wang et al. (2018) found that administering 2% sevoflurane over 3 h of ESLP resulted in reduced inflammation with lower levels of TNF-α along with less weight gain and perivascular edema compared to ESLP without sevoflurane [166]. In similar studies of canine ESLP, β-adrenoreceptor agonists administered during ESLP have demonstrated improved lung function as assessed by increased PaO2 and compliance [167,168]. These findings suggest that sevoflurane and β-adrenoreceptor agonists have the potential to protect lung function and can help recondition damaged lungs prior to transplantation.

### 4.7. The Future of ESLP

As outlined above, ESLP is the way forward to improve lung transplant outcomes: increase the number of transplantations performed each year, expand the geographic distance over which donor lungs can be retrieved, and provide a vehicle to deliver therapeutics. ESLP has proven instrumental for research into the cause and treatment of LIRI in clinical and laboratory settings [169,170,171]. For patients, it has improved donor lung availability and quality, which translates into more lives saved.

## 5. Conclusions

Lung transplantation for end-stage lung disease is limited by an inadequate number of suitable donor lungs for waitlisted patients and technological advancement can help address this discrepancy. Approximately 20% of lungs offered for donation are accepted for transplantation with the remainder deemed unacceptable due to stringent acceptance criteria, which contributes to high-waitlist mortality.

LIRI principally targets the pulmonary endothelium and is the main cause of PGD following lung transplantation. LIRI results in self-perpetuating ROS generation and cytokine signaling cascades, immune system activation with neutrophil infiltration of the lung parenchyma, ionic imbalances that alter cell structure and function causing apoptosis and necrosis, complement and platelet activation causing microcirculatory blood flow heterogeneity and dysfunction, and endothelial cell inflammation leading to gap formation with increased permeability. The culmination is that of pulmonary edema and impaired oxygenation with deleterious systemic effects.

The current standard for organ preservation is CSP; however, ESLP offers an improved approach by ventilating and perfusing the lungs continuously for a more physiologic preservation strategy with real-time feedback. ESLP represents the greatest leap forward in lung transplantation since the introduction of anti-rejection therapies. This technology has already begun to increase the donor pool by expanding the geographic catchment of donors, reconditioning extended criteria donors, and enabling the application of novel treatment modalities, such as stem cell and gene therapies, that previously lacked a viable route into the transplant algorithm.

NPV-ESLP is a unique form of ESLP that uses a more physiologic ventilation strategy compared to other platforms. A number of significant achievements have been made with the use of this technology in recent years culminating in a successful clinical trial involving extended criteria donor lungs. Many fascinating opportunities remain to further refine NPV-ESLP methodology to evaluate, recondition, and preserving donor lungs.

## Figures and Tables

**Table 1:** ISHLT PGD grading [44].

Grade	P/F Ratio	Chest X-ray
0	>300	Normal
1	>300	Diffuse Allograft Infiltrates
2	200–300	Diffuse Allograft Infiltrates
3	<200	Diffuse Allograft Infiltrates

## Data Availability

Not applicable.
